# Assessment of unsuppressed viral load and its determinants among pregnant and breastfeeding women living with HIV in the prevention of vertical transmission programme in Bugesera District, Rwanda, 2023

**DOI:** 10.1186/s12884-026-09822-7

**Published:** 2026-08-14

**Authors:** Didier Ndabana, Bibiane Uwamahoro, Judith Mukamurigo

**Affiliations:** 1 Nyamata Level 2 Teaching Hospital, TBHIV, Kigali, +250 Rwanda; 2 University of Rwanda, CHMS, Kigali, +250 Rwanda

**Keywords:** Viral load unsuppression, pregnant women, breastfeeding, HIV, Prevention of vertical transmission of HIV

## Abstract

**Background:**

Prevention of vertical transmission (PVT) of HIV programs are designed to prevent the transmission of HIV during pregnancy and breastfeeding. Nevertheless, attaining viral suppression among mothers living with HIV remains a major challenge, especially in resource-constrained settings like Bugesera District, Rwanda. This study aim to determine the prevalence and determinants of unsuppressed viral load (VL) among pregnant and breastfeeding women living with HIV enrolled in the PVT of HIV program as of 2023.

**Methods:**

An analytical cross-sectional study using retrospective record review was carried out using secondary data from 508 pregnant and breastfeeding women living with HIV enrolled in the PVT of HIV services in 15 health centres in Bugesera District between January 2023 and December 2023, Data were extracted from patient records and analysed using descriptive statistics, bivariate analysis, and multivariate logistic regression to identify factors associated with an unsuppressed VL (≥ 200 copies/mL).

**Results:**

Among the 508 pregnant and breastfeeding women living with HIV enrolled in the PVT of HIV services, the prevalence of unsuppressed VL was 8.5% (95% CI: 6.3–11.2). factors associated with unsuppressed VL were mental health problems (adjusted odds ratio [aOR] = 4.7, 95% CI: 2.3–9.5, *p* < 0.001), low attendance to antenatal care (ANC) (< 4 visits) (aOR = 2.2, 95% CI: 1.1–4.7, *p* = 0.025), a history of treatment interruption (aOR = 2.4, 95% CI: 1.1–4.9, *p* = 0.015) and being married or cohabiting (aOR = 2.0, 95% CI: 1.0–4.1, *p* = 0.035).

**Conclusion:**

The unsuppression viral load rate among women living with HIV in Bugesera District was high. This study highlights the need of strengthening psychosocial support, increase attendance of antenatal care consultations and improve antiretroviral (ART) retention in the PVT of HIV program, particularly among women who are partnered.

## Introduction

Vertical transmission of HIV remains an important public health challenge in sub-Saharan Africa, where HIV prevalence is high [1]. Vertical transmission of HIV is the transmission of HIV from a woman living with HIV to her baby. This can occur in utero, at delivery, or via breastfeeding and is most triggered by high HIV viral load in plasma of the infected mother [2].

Due to the need to eliminate vertical transmission of HIV and the importance of protecting the health of both HIV-exposed children and the pregnant or breastfeeding women living with HIV, prevention of vertical transmission (PVT) of HIV remain pivotal to these efforts [3].In 2013, the World Health Organization (WHO) recommended that all pregnant or breastfeeding women living with HIV should be started on life-long antiretroviral therapy (ART). In line with both WHO guidance and the guidelines for HIV Prevention, Treatment and Care in Rwanda, HIV-exposed infants are provided with postnatal antiretroviral prophylaxis from birth until six weeks of age to reduce the risk of vertical transmission [4, 5]. This guideline has been implemented to improve the health of women living with HIV(WLHIV) during pregnancy and lactation, but sustaining viral load (VL) suppression in pregnant and breastfeeding women living with HIV is still a major public health challenge, particularly, in resource limited settings [5]. Higher levels of VL suppression are important to improve the health of individuals living with HIV and to stop further transmission of HIV [6].

Unsuppressed VL (defined by the national guideline as ≥ 200 copies/mL of plasma HIV RNA copies) increases the risk of Vertical transmission of HIV and is a marker of ART failure [4]. In 2023, an estimated 1.4 million children aged ≤ 15 years were living with HIV, and 120,000 new HIV infections had occurred globally; these were primarily due to vertical transmission and in particular in Sub-Saharan Africa [7].

Maternal VL in pregnancy and lactation is an important measure of ART effectiveness and risk of vertical transmission of HIV [8]. Routine VL testing, recommended by WHO since 2016, enables the early identification of unsuppressed VL followed by corrective action. In sub-Saharan Africa, however, unsuppressed VL rates range from 6.1% to 15.4%, while VL testing uptake in pregnant women on lifelong ART remains between 35% and 50% [5, 11]. Maternal VL plays a significant role in HIV transmission to the foetus, emphasizing the importance of better VL monitoring in PVT of HIV programs.

In low- and middle-income countries such as Rwanda, breastfeeding is a common practice, and in the absence of effective antiretroviral therapy, the risk of vertical transmission of HIV during breastfeeding remains high [9]. Transmission can occur 20–45% of the time, with 5–10% of transmission occurring in pregnancy, 10–20% in delivery and 5–20% in breastfeeding [10].

The ART coverage among pregnant women living with HIV in Rwanda was 96.9% in 2019, and 99% of pregnant women attended at least one antenatal care visit, with PVT of HIV services offered in 98% of health facilities [11]. Since 2012, all pregnant women living with HIV in Rwanda are on ART regardless of CD4 count, and exposed infants are exclusively breast fed for up to six months while protected by ART. Mothers continue ART beyond this period as well. National data from Health Management Information System (HMIS) (July 2021–June 2022) showed that uptake of Option B + was associated with a reduction in the vertical transmission of HIV rate to 1.3% at 24 months across the cohort of HIV-exposed infants [12]. While there have been significant improvements, vertical transmission continues to occur, indicating that maintaining maternal viral load suppression during both pregnancy and during the breastfeeding phase is key to ending such transmissions.

Multiple studies have found that a number of factors contribute to unsuppressed VL including sociodemographic factors (younger age, marital status, low frequency of antenatal care visits); clinical factors (late ART initiation, ART regimen, clinical stage, coinfections); and healthcare system-related factors (availability of consumables, sample transportation issues, infrequent VL monitoring, geographic barriers, staff-to-patient ratio [8, 13, 14]. Tackling these barriers is vital to enhancing health outcomes, and meeting HIV elimination targets, both nationally, and globally. Despite the expansion of prevention of vertical transmission of HIV services in Bugesera District, there is limited evidence on viral load suppression and its determinants among pregnant and breastfeeding women living with HIV.

This study therefore aims to determine the prevalence and determinants of unsuppressed VL among pregnant and breastfeeding women living with HIV enrolled in the PVT of HIV program in Bugesera District, Rwanda, in 2023. The findings from this study will provide critical input to PVT of HIV interventions and contribute to efforts toward elimination of vertical transmission of HIV, while contributing to improved pregnant women and child health outcomes in Rwanda and similar settings.

## Methods

### Study settings

The study was conducted in all health centers providing PVT of HIV services located in Bugesera District, Rwanda. There are 15 health centers offering PVT of HIV services in the district: Gashora, Gakurazo, Gihinga, Juru, Kamabuye, Mayange, Mareba, Mwogo, Ngeruka, Ntarama, Nyamata, Nyarugenge, Nzangwa, Rilima and Ruhuha.

### Study design and period

We conducted an analytical cross-sectional study using retrospective record review of pregnant and breastfeeding women living with HIV who were enrolled in the PVT of HIV program in Bugesera District, Rwanda in 2023. The study was conducted from July 12 to 28, 2024 to determine the prevalence of unsuppressed viral load and its determinants. This study used secondary data extracted from routine PVT of HIV programme records maintained by health facilities providing PVT of HIV services in Bugesera District.

### Study population

The study population consisted of pregnant and breastfeeding women living with HIV enrolled in the PVT of HIV program in Bugesera District between January-December 2023.

#### The inclusion criteria were as follows:

Pregnant and breastfeeding women living with HIV within the PVT of HIV program during the study period. Viral loads for all pregnant women and breastfeeding women living with HIV on ART were measured at baseline and 6 months and recorded in their medical records.

#### Exclusion criteria

We excluded (1) Pregnant and breastfeeding women living with HIV newly diagnosed with HIV within six months prior to screening, (2) pregnant women outside the study period (January–December 2023), and (3) those with incomplete medical records or missing viral load test results.

### Sample size determination and sampling technique

An exhaustive sample of Pregnant and breastfeeding women living with HIV was taken. All records during the study period were analysed. Therefore, this study included all eligible medical records of Pregnant and breastfeeding women living with HIV who were enrolled in PVT of HIV program in Bugesera District between January-December 2023 and met the inclusion criteria. During this period, the number of records identified from medical records was 569. The entire Bugesera District health centers who provide PVT of HIV services (a total of 15) were included in the study to provide a comprehensive coverage of the study area. The number of records included from each health centre therefore corresponded to the total number of pregnant and breastfeeding women who were enrolled in the PVT of HIV program in Bugesera District in 2023.

Data were extracted from multiple routine data sources, including ART treatment files for women living with HIV on antiretroviral therapy, antenatal care (ANC) registration books for information for ANC visits, and maternity registries for record related to deliveries. Additional sources included the ART register, mother‒child couple follow-up registers in the PVT of HIV program, and viral load books documenting viral load test results.

**Fig. 1:**
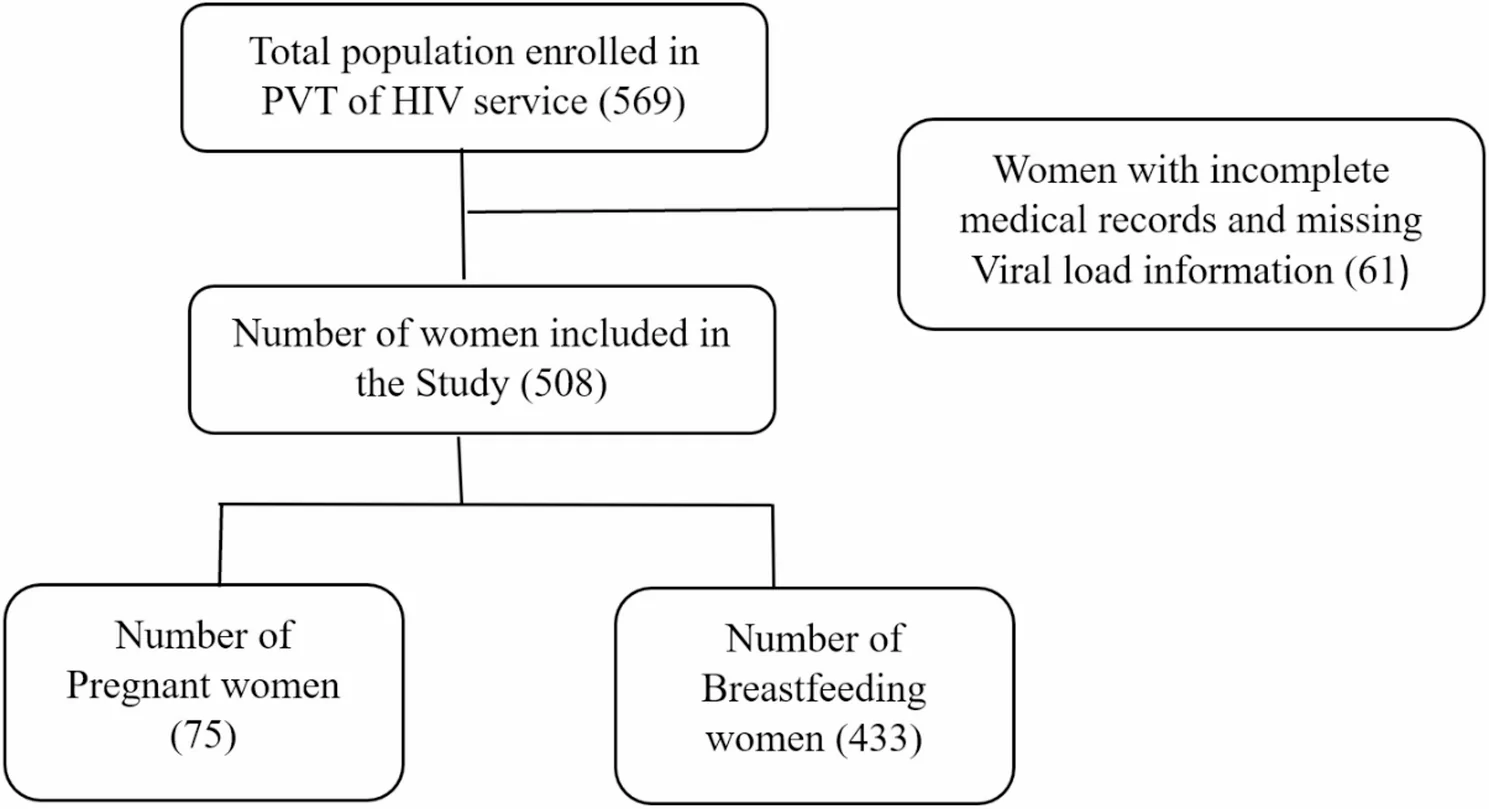
Flow Chart of Study Participant selection

From the total records of pregnant and breastfeeding women living with HIV enrolled in the PVT of HIV program in Bugesera District during 2023, 61 records were excluded due to incompleteness or failure to meet the predefined exclusion criteria, as illustrated in the cascade in (Fig. 1).

### Variables and measurements

#### Dependent variable

Unsuppressed Viral Load: The main outcome of interest is the presence of an unsuppressed viral load, defined according to the Rwandan national HIV guidelines as a viral load ≥ 200 copies/mL [4].

#### Independent variables

Gravidity, Marital status, Profession, Age, date of HIV positivity, Timing of ART initiation, Gestational Age, Health facility ownership, ANC attendance, Place of birth quality of adherence to ART, ART regimen, Partner’s HIV status, baseline CD4 count, Time on ART, History of ART regimen switch, History of clinical failure, History of treatment interruption (yes, no), HIV stigma, Partner tested for HIV, Drug side effect, Drug interaction, Comorbidity. WHO Stage Mental health screening status, BMI.

### Data collection tools and procedures

Demographics, laboratory VL results and participants’ clinical information were obtained from ART files for Pregnant and breastfeeding women living with HIV receiving antiretroviral therapy, complemented by viral load books, antenatal care (ANC) registration books, Mother‒Child Couple Follow-up registers and maternity registers via the data extraction sheet designed in Epi Info 7. Data abstraction was performed by two trained data collectors from the University of Rwanda, School of Public Health, supported by Nurses working in the PVT of HIV program who are knowledgeable about the program.

All charts containing detailed information regarding VL in pregnant and breastfeeding women were reviewed. When there were incomplete data from one tool, the data collectors tried to obtain data from different available sources (such as the EMR system). Participants without inclusion criteria were excluded during data collection.

### Data management and quality assurance

Data integrity was guaranteed by creating appropriate data collection tools. The required data collectors were trained before data collection, with respect to the study’s goal, data extraction techniques, data collection tools, data sources and variables. A supervisor ensured that no data were missing throughout the data collection time. The mentor and supervisor oversaw every stage of the activities related to the research. The data collection tool was pretested at Nyamata Health Centre to ensure its reliability and validity before use.

Following data entry, frequency, cross tabulation, sorting, and listing were used to clean the data and check for outliers and missing values. The data from the study were stored on a protected computer, and the data were collected with the support of key staff who were very well aware of the service and who contributed to the follow-up of pregnant and breastfeeding women living with HIV in the PVT of HIV.

### Data analysis

The prevalence of unsuppressed viral load and sociodemographic, clinical, and related factors were reported using descriptive statistics.

A binary variable created for independent variables was coded with “1 = Yes” and “0 = No” during Data analysis, where 1 represented the presence of a factor, while the 0 was used as a reference category by default.

Bivariate analysis was performed to evaluate the associations between independent variables (sociodemographic and clinical factors) and dependent variable (unsuppressed viral load). A multivariable logistic regression model was used to determine factors independently associated with unsuppressed viral load. The model included variables identified as significant in bivariate analysis and controlled for potential confounders, and a significant association was indicated at a p value of 0.05.

Collinearity was assessed by means of a Pearson correlation matrix. The variables with strong collinearity in the model of logistic regression were eliminated from the model in data analysis to exclude the collinearity effect in regression model. The data were analysed using STATA 17.

### Ethical considerations

Prior to the commencement of the study, the study protocol version was finalized and ethical approval was obtained from the IRB of the University of Rwanda, College of Medicine, and Health Sciences (Ref. No. CHMS/IRB456/2024) and access to patients’ files was granted by the Nyamata Level 2 Teaching Hospital representing the 15 health centres involved in the study (Ref. No.267/HN/2024). The review committee waived the necessity for informed consent, as there were secondary data retrieved from PVT of HIV and the names were coded, no personal identifiers (e.g. name, or address) were obtained. Patients were not harmed, and confidentiality was maintained. All methods were conducted in compliance with relevant procedures and protocols.

## Results

### Characteristics of the study participants

Overall, 508 women were studied in this study. The median age was 30 years (IQR: 26–35), with the most prevalent age group being 25–34 years (53.0%). The majority of participants (85.2%) were breastfeeding during the survey. With respect to marital status, 57.09% were single The study revealed that most participants (77.4%) were engaged in agricultural activities. With respect to healthcare services, 86.2% of the respondents were followed in government health centers. Regarding the place of delivery, 71.6% delivered at a health centre and a total of 49.0% of the participants had low antenatal care (ANC) attendance, defined as fewer than four visits during pregnancy.

Most of the participants had achieved viral suppression, with 91.5% (95% CI: 88.7–93.6) of participants having suppressed viral loads, and 8.5% (95% CI: 6.3–11.2) having unsuppressed viral loads. In terms of treatment history, almost half (46.6%) of the participants reported treatment interruption (i.e., have not visited the hospital or clinic for ART for more than one month). With regards to mental health, 22.05% of the participants showed signs of mental health problems. While adherence to ART was generally high (87.6%), 45.0% of participants did not know the HIV status of their partners. The vast majority of participants were on 1st line ART regimens (99.0%). The majority of the participants had a Body Mass index (BMI) ≥ 18.5(94.49%) (Table 1).

### Prevalence of unsuppressed viral load

The prevalence of unsuppressed viral load (VL) was determined as the proportion of participants with a VL result ≥ 200 copies/mL among those with a documented result after at least six months of antiretroviral therapy (ART). Based on this definition, 43 out of 508 participants were found to have an unsuppressed viral load. This corresponds to a prevalence of 8.5% (95% CI: 6.3–11.2). Conversely, the remaining 91.5% of participants had achieved viral suppression. This calculation is derived from the total eligible study population (*N* = 508) identified through the exhaustive sampling of the PVT of HIV programme records in Bugesera District for 2023.

**Table 1:** Participants characteristics

Variable	Frequency	Percentage
Maternal status		
Pregnant	75	14.8
Breastfeeding	433	85.2
Age years, median (IQR)	30 (26–36)	
Age group		
18–24 years	91	17.9
25–34 years	269	53.0
35 and above	148	29.1
Marital status		
Married/cohabiting	218	42.9
Single	290	57.1
Gravidity		
1	110	21.7
2–3	219	43.1
4–5	157	30.9
6 and above	22	4.3
Profession		
Agriculture	393	77.4
Services	19	3.7
Other	96	18.9
Gestational age		
Preterm	21	4.1
Term	487	95.9
Health facility ownership		
Government	438	86.2
Faith-based (mission) & other	70	13.8
ANC attendance		
Full attendance (≥ 4 visits)	259	51
Low attendance (< 4 visits)	249	49
Place of Delivery		
Health centre	364	71.6
Hospital	124	24.4
Home	13	2.6
Another place	7	1.4
BMI		
BMI < 18.5	28	5.5
BMI ≥ 18.5	480	94.5
Viral load suppression (95% CI)		
Suppressed	465	91.5 (88.7–93.6)
Unsuppressed	43	8.5 (6.3–11.2)
History of treatment interruption		
Yes	237	46.7
No	271	53.3
History of clinical failure		
Yes	9	1.8
No	499	98.2
Mental health screening status		
Positive	112	22.1
Negative	396	77.9
Quality of adherence		
Good	445	87.6
Poor	36	7.1
Fair	27	5.3
History of ART regimen switch		
No	437	86
Yes	71	14
Partner’s HIV status		
Negative	114	22.4
Positive	165	32.5
Unknown	229	45.1
Drugs interaction		
Yes	6	1.2
No	502	98.8
Comorbidity		
Yes	5	1
No	503	99
ART start year		
2008 and under	28	5.5
2009–2013	58	11.4
2014–2018	105	20.7
2019–2023	317	62.4
ART regimen		
First-line	503	99
Second-line	5	1
WHO stage		
Stage 1	490	96.5
Stage 2	18	3.5

*BMI* Body Mass Index, *IQR* Interquartile Range, *ANC* Antenatal Care, *ART* Antiretroviral Treatment, *WHO* World Health Organization

*Confidence Interval (95% CI)

### Bivariate analysis of factors associated with unsuppressed viral load

Bivariate analysis revealed that individuals with mental health problems had 4.3 times greater odds of viral load unsuppression than did those without mental health problems (95% CI: 2.3–8.2, *p* < 0.001), and individuals with low antenatal care (ANC) attendance (< 4 visits) had 2.9 times greater odds of viral load unsuppression than did those with full ANC attendance (95% CI: 1.4–5.8, *p* = 0.002). Individuals with a history of treatment interruption (missing ART for more than one month) had 2.8 times greater odds of viral load unsuppression than those without such a history (95% CI: 1.4–5.6, *p* = 0.002). Individuals with a history of clinical failure had 9.4 times greater odds of viral load unsuppression than did those without clinical failure (95% CI: 2.4–36.5, *p* = 0.001). Compared with those without drug interactions, individuals who experienced drug interactions during ART had 23.7 times greater odds of viral load unsuppression (95% CI: 4.2–133.0, *p* < 0.001). Individuals in WHO Stage 2 had 3.3 times greater odds of viral load unsuppression than those in WHO Stage 1 did (95% CI: 1.0–10.5, *p* = 0.043). Compared with single individuals, married or cohabiting individuals had 1.9 times greater odds of viral load unsuppression (95% CI: 1.0–3.6, *p* = 0.038). Compared with those with term births, individuals with preterm births (< 28 weeks of gestation) had 3.6 times greater odds of viral load unsuppression (95% CI: 1.2–10.6, *p* = 0.015) (Table 2).

**Table 2:** Bivariate analysis of factors associated with unsuppressed viral load

Variables	Suppressed *n* (%)	Unsuppressed *n* (%)	COR	95% CI	*P* value
Maternal status					
Breastfeeding	397 (78.1)	36 (7.1)	0.9	(0.4–2.1)	0.77
Pregnant	68 (13.4)	7 (1.4)	Ref.		
Age group					
18–24 years	82 (16.1)	9 (1.8)	1.5	(0.5–3.8)	0.387
25–34 years	245 (48.2)	24 (4.7)	1.3	(0.6–2.9)	0.441
35-and above	138 (27.2)	10 (2.0)	Ref.		
Marital status					
Married/Cohabitant	193 (38.0)	25 (4.9)	1.9	(1.0-3.6)	0.038*
Single	272 (53.5)	18 (3.5)	Ref.		
Gravidity					
Primigravida	93 (18.3)	17 (3.3)	1.8	(0.3–8.5)	0.443
2_3	202 (39.8)	17 (3.3)	0.8	(0.1–3.9)	0.826
4_5	150 (29.5)	7 (1.4)	0.4	(0.0-2.4	0.362
6 and above	20 (3.9)	2 (0.4)	Ref.		
Profession					
Agriculture	364 (71.7)	29 (5.7)	0.5	(0.2–1.1)	0.109
Services	17 (3.3)	2 (0.4)	0.8	(0.1-4.0)	0.81
Other	2 (0.4)	2 (0.4)	Ref.		
Gestational age					
Pre term	16 (3.1)	5 (1.0)	3.6	(1.2–10.6)	0.015*
Term	449 (88.4)	38 (7.5)	Ref.		
Health facility ownership					
Government	401 (78.9)	37 (7.3)	1	(0.4–2.5)	0.972
Faith-based (mission)&other	64 (12.6)	6 (1.2)	Ref.		
ANC Visit (Full attendance)					
Low attendance	247 (48.6)	12 (2.4)	2.9	(1.4–5.8)	0.002*
Full attendance	218 (42.9)	31 (6.1)	Ref.		
Place of birth					
Health centre	342 (67.3)	22 (4.3)	0.3	(0.0-1.6)	0.194
Hospital	107 (21.1)	17 (3.3)	0.8	(0.1–4.2)	0.868
Home	11 (2.2)	2 (0.4)	Ref.		
Another place	5 (1.0)	2 (0.4)	2.2	(0.0-0.8)	0.488
History of treatment interruption					
Yes	207 (40.7)	30 (5.9)	2.8	(1.4–5.6)	0.002*
No	258 (50.8)	13 (2.6)	Ref.		
History of clinical failure					
Yes	28 (5.5)	43 (8.5)	9.43	(2.4–36.5)	0.001*
No	437 (86.0)	0 (0.0)	Ref.		
Mental health problem					
Yes	90 (17.7)	22 (4.3)	4.3	(2.3–8.2)	< 0.001*
No	375 (73.8)	21 (4.1)	Ref.		
Partner’s HIV status					
Negative	101 (19.9)	13 (2.6)	Ref.		
Positive	146 (28.7)	19 (3.7)	1	(0.4–2.1)	0.977
Unknow	218 (42.9)	11 (2.2)	0.3	(0.1–0.9)	0.028*
Drugs interaction					
Yes	2 (0.4)	4 (0.8)	23.7	(4.2–133)	< 0.001*
No	463 (91.1)	39 (7.7)	Ref.		
Comorbidity					
Yes	2 (0.4)	3 (0.6)	17.36	(2.8 -106.9)	0.002*
No	463 (91.1)	40 (7.9)	Ref.		
ART Start year					
2008 & under	24 (4.7)	4 (0.8)	Ref.		
2009_2013	56 (11.0)	2 (0.4)	0.21	(0.0-1.2)	0.087
2014_2018	92 (18.1)	13 (2.6)	0.8	(0.2–2.8)	0.789
2019_2023	293 (57.7)	24 (4.7)	0.4	(0.1–1.5)	0.221
WHO Stage					
Stage 1	451 (88.8)	39 (7.7)	Ref.		
Stage 2	14 (2.8)	4 (0.8)	3.3	(1.0 -10.5)	0.043*

*ART* Antiretroviral therapy, *ANC* Antenatal Care, *Ref* Reference, *COR* Crude odds ratio

*Significant p-values (< 0.05)

### Multivariate analysis of factors associated with unsuppressed viral load

As part of the data-cleaning process, variables with extremely low frequencies in their categories, such as WHO stage, comorbidities, drug interactions, and history of clinical failure, were excluded prior to inclusion in the multivariate analysis. These variables were removed because categories with few observations can result to broad confidence intervals, which usually leads to instability or unreliability in estimating regression coefficients. Including these variables in the final model can produce misleading interpretations.

The quality of adherence was excluded from the multivariable model because of collinearity with the history of treatment interruption. Additionally, variables such as history of ART regimen switching, ART regimen, and BMI were removed because they showed no variability and were not meaningful in the statistical analysis. Variables with p values > 0.5, including those related to the study group stratification, were also excluded from the adjusted model. After controlling for potential confounders using a multivariate logistic regression model, mental health problems with an adjusted odds ratio (aOR) of 4.7 (95% CI: 2.3–9.5), *p* < 0.001. Low antenatal care attendance (< 4 visits): aOR = 2.2 (95% CI: 1.1–4.7), *p* = 0.025. History of treatment interruption: aOR = 2.4 (95% CI: 1.1–4.9), *p* = 0.015. Being married or cohabiting: aOR = 2.0 (95% CI: 1.0–4.1), *p* = 0.035, was significantly associated with an unsuppressed viral load (Table 3).

**Table 3:** Multivariate Analysis of Factors Associated with Unsuppressed Viral Load

Variables	Suppressed	Unsuppressed	COR (95%CI)	*P* value	AOR (95%CI)	*P* value
Marital status						
Married/Cohabitant	193 (38.0)	25 (4.9)	1.9(1.0-3.6)	0.038	2.0(1.0-4.1)	0.035*
Single	272 (53.5)	18 (3.5)	Ref.		Ref.	
Gestational age						
Pre term	16 (3.1)	5 (1.0)	3.6(1.2–10.6)	0.015	2.2(0.6–8.1)	0.204
Term	449 (88.4)	38 (7.5)	Ref.			
ANC Visit						
Full attendance	247 (48.6)	12 (2.4)	Ref.			
Low attendance	218 (42.9)	31 (6.1)	2.9(1.4–5.8)	0.002	2.2(1.1–4.7)	0.025*
History of treatment interruption						
Yes	207 (40.7)	30 (5.9)	2.8(1.4–5.6)	0.002	2.4(1.1–4.9)	0.015*
No	258 (50.8)	13 (2.6)	Ref.		Ref.	
Mental health problem						
Yes	90 (17.7)	22 (4.3)	4.3(2.3–8.2)	< 0.001	4.7(2.3–9.5)	< 0.001*
No	375 (73.8)	21 (4.1)	Ref.			

*AOR* Adjusted odds Ratio, *COR* Crude Odds Ratio

*Significant *p*-values (< 0.05)

## Discussion

This study evaluated the prevalence and determinants of unsuppressed viral load (VL) among pregnant-women and breastfeeding-women living with HIV enrolled in the PVT of HIV program in Bugesera District, Rwanda, 2023. The results provide important insights into key barriers to attaining universal VL suppression and enrich the consideration of similar populations of PLHIV across sub-Saharan Africa. The overall prevalence of these unsuppressed VL was 8.46% (95% CI: 6.3–11.2); thus, 91.54% of participants had viral suppression ≥ 6 months after initiating ART. Although this level of viral suppression is relatively high, it remains below the 95% target set by the Joint United Nations Programme on HIV/AIDS(UNAIDS) [15], indicating that additional efforts are required to achieve optimal treatment outcomes. The prevalence in Bugesera District appears low in comparison to results observed in other sub-Saharan African countries, for which the prevalence of unsuppressed VL ranges from 11.8% among pregnant and breastfeeding women in Ethiopia, to 62.1% in Côte d’Ivoire [16]. This finding emphasizes the progress achieved in upholding PVT of HIV in Rwanda via lifelong ART through Option B+ implementation and routine VL monitoring.

VL were unsuppressed in a much higher proportion of those with mental health problems than those without mental health problems. According to the WHO, Mental health is defined as “a state of mental well-being that enables people to cope with the stresses of life, realize their abilities, learn well and work well, and contribute to their community [17]. In Bugesera District, anxiety, depression, and other mental health disorders are prevalent among pregnant and breastfeeding women, especially among people living with HIV. These difficulties could arise from several human conditions: partner conflict on sharing HIV status, HIV stigma, psychosocial stress when living with HIV (and insufficient psycho-social support provided by healthcare workers) [18–20]. Loneliness and low social support have been implicated for depressive symptoms in pregnancy [21]. There is also evidence that depression may hinder the immune system [22], the exact mechanisms of which remain unclear for now. These effects on immune function can enhance the challenge to achieve VL suppression. The current study findings are consistent with earlier studies like [23], which demonstrate that psychological distress, depression, and stigma have been detrimental to ART adherence. To address these mental health problems, we recommend the systematic integration of mental health screening and basic psychological counselling into routine PVT services. A significant intervention would be the implementation of structured “Mentor Mother” programmes, where experienced WLHIV provide peer-led psychosocial support. The primary benefit of this model is the reduction of stigma and the improvement of status sharing within couples, which directly correlates with better adherence. However, a potential drawback is the requirement for additional resources for training and the need for formal clinical supervision to ensure the quality of peer-led counselling.

Higher odds of unsuppressed VL were observed for ANC attendance lower than WHO optimal levels. These findings are corroborated by South Africa, Uganda and Kenya studies that have shown that the likelihood of healthier maternal outcomes increased with more frequent ANC visits as they offer opportunities for unique adherence counselling, monitoring ART, and timely interventions [8, 24]. Rwanda has adopted the WHO recommendation of at least four ANC visits, preferably the first visit initiated within the first 12 weeks of gestation [25]. Low ANC attendance in this context means less than four visits during pregnancy. Other Rwandan evidence indicates that cultural beliefs and social norms in specific contexts lead to postponed or inappropriate pregnancy sharing HIV status, thus affecting ANC attendance [26]. Low attendance at ANC can decrease VL monitoring frequency and limit opportunities to address adherence barriers, which are critical for achieving viral suppression. Our results highlight the need for interventions to strengthen early and consistent ANC attendance, Potential interventions to improve attendance include community-based outreach and the use of digital health reminders to notify women of upcoming appointments. While these tools are highly effective for scheduling, they may be limited by inconsistent mobile phone access or literacy levels among the most vulnerable populations.

Having a history of treatment interruption was significantly correlated with unsuppressed VL. This finding is consistent with studies from other sub-Saharan African settings, in which ART interruptions resulting from logistical challenges, stigma, non-sharing HIV status to partners or economic constraints are strongly associated with ART failure and unsuppressed VL [27, 28]. Results indicate that the expansion of Differentiated Service Delivery (DSD) models through community-based ART refill groups can significantly reduce the travel burden and time costs women experience associated with accessing ART refills. It should be emphasized, however, that this intervention requires a reliable tracking method to confirm that women complete their necessary clinical laboratory assessments for VL monitoring.

Marital status may affect outpatient adherence to antiretroviral therapy (ART) and viral load (VL) suppression in women, but the evidence is mixed. Some studies suggest that women who are married or cohabiting have higher ART adherence than single women. For instance, a study has indicated that adherence is 70.8% for married participants compared to 51.6% for single participants [29]. In contrast, another study concluded that being married was associated with lower adherence due to the potential for non-sharing of HIV-positive status to partners [8, 30]. These contrasting results underscore the nuanced relationship between marital status, ART adherence, and VL suppression [8]. In Bugesera, partner-related factors, such as stigma, lack of support and failure to disclose HIV status may negatively affect adherence and result in unsuppressed viral load. This context may differ from that of other regions, potentially explaining the divergent findings. Evidence suggests that individuals living with HIV are reluctant to disclose their HIV status to sexual partners out of fears of abandonment. Nondisclosure, however, is associated with poor adherence to ART and an increased risk of unsuppressed viral load [16, 31, 32].

Our study could not demonstrate similar associations between age, gravidity, parity, or gestational age and unsuppressed viral load (VL). This finding corresponds with a study done in Mutare, Zimbabwe that found these factors were not predictors of VL sample collection in pregnant women living with HIV [13].

### Limitations

The retrospective nature of the data used in this study limited its ability to analyse a range of socioeconomic and contextual factors (e.g., income, education, household context, and health system-related factors). Furthermore, there was no data available on CD4 counts limiting insight into participants’ immunological status. In terms of statistical robustness, although the overall sample size was relatively large, the number of participants with unsuppressed viral load was small, which may have limited the statistical power to detect small differences between groups. As a result, some potentially important associations may not have reached statistical significance. The cross-sectional design limits the establishment of causal relationships between identified determinants and unsuppressed VL. Future studies should explore longitudinal designs to assess the temporal relationships between core determinants and VL outcomes. Furthermore, examining community-level factors that facilitate or hinder ART adherence can further elucidate factors responsible for unsuppressed VL. Most importantly the results from this study are limited to Bugesera District and they might not be representative of the whole country. However, these lessons will help guide local efforts to enhance VL suppression in analogous settings.

## Conclusion

The prevalence of unsuppressed viral load among pregnant and breastfeeding women living with HIV in Bugesera District was estimated at 8.5%, high than the national target of < 5% and below the 95% viral suppression target set by the Joint United Nations Programme on HIV/AIDS. Notably, the significant factors associated with viral unsuppression included mental health problems, poor attendance at ANC consultations, having a history of treatment interruption, and living with a partner. These findings highlight persistent gaps in achieving optimal viral suppression despite the scale-up of prevention of vertical transmission services and lifelong antiretroviral therapy.

Addressing these gaps requires integrated, patient-centred interventions. Strengthening the integration of mental health screening and care within HIV services including structured psychosocial support and peer-led approaches such as mentor mother programmes may improve adherence and viral suppression. In addition, interventions to enhance early and consistent antenatal care attendance, strengthen adherence counselling, and reduce treatment interruption are essential.

While these interventions have clear potential benefits, including improved maternal health outcomes and a reduced risk of vertical transmission, their implementation may require additional resources, trained personnel, and sustained programme coordination, particularly in resource-limited settings. Therefore, context-specific and scalable approaches are needed.

If not addressed, these factors may hinder progress towards national and global targets for ending vertical transmission of HIV and improving maternal and child health outcomes. Strengthening integrated service delivery within prevention of vertical transmission programmes remains critical to achieving sustained viral suppression among pregnant and breastfeeding women living with HIV.

## Data Availability

The datasets used in this study are available from the corresponding author on reasonable request.

## Abbreviations

ART: Antiretroviral therapy
PVT: Prevention of the vertical transmission
VL: Viral load
ANC: Antenatal care
BMI: Body mass index
UNAIDS: United Nations Programme on HIV/AIDS
RNA: Ribonucleic Acid
WLHIV: Women Living with HIV
